# L-Lactate Electrochemical Biosensor Based on an Integrated Supramolecular Architecture of Multiwalled Carbon Nanotubes Functionalized with Avidin and a Recombinant Biotinylated Lactate Oxidase

**DOI:** 10.3390/bios14040196

**Published:** 2024-04-16

**Authors:** Alejandro Tamborelli, Michael López Mujica, Marilla Amaranto, José Luis Barra, Gustavo Rivas, Agustina Godino, Pablo Dalmasso

**Affiliations:** 1 CIQA, CONICET, Departamento de Ingeniería Química, Facultad Regional Córdoba, Universidad Tecnológica Nacional, Maestro López esq. Cruz Roja Argentina, Córdoba 5016, Argentina; alejandrotambrelli@gmail.com; 2 INFIQC, CONICET-UNC, Departamento de Fisicoquímica, Facultad de Ciencias Químicas, Universidad Nacional de Córdoba, Ciudad Universitaria, Córdoba 5000, Argentina; mlopezmujica@unc.edu.ar; 3 CIQUIBIC, CONICET-UNC, Departamento de Química Biológica Ranwel Caputto, Facultad de Ciencias Químicas, Universidad Nacional de Córdoba, Ciudad Universitaria, Córdoba 5000, Argentina; marilla.amaranto@unc.edu.ar (M.A.); jose.luis.barra@unc.edu.ar (J.L.B.)

**Keywords:** lactate, electrochemical biosensor, multiwalled carbon nanotubes, avidin, biotinylated lactate oxidase, recombinant proteins, supramolecular recognition

## Abstract

L-Lactate is an important bioanalyte in the food industry, biotechnology, and human healthcare. In this work, we report the development of a new L-lactate electrochemical biosensor based on the use of multiwalled carbon nanotubes non-covalently functionalized with avidin (MWCNT-Av) deposited at glassy carbon electrodes (GCEs) as anchoring sites for the bioaffinity-based immobilization of a new recombinant biotinylated lactate oxidase (bLOx) produced in *Escherichia coli* through *in vivo* biotinylation. The specific binding of MWCNT-Av to bLOx was characterized by amperometry, surface plasmon resonance (SPR), and electrochemical impedance spectroscopy (EIS). The amperometric detection of L-lactate was performed at −0.100 V, with a linear range between 100 and 700 µM, a detection limit of 33 µM, and a quantification limit of 100 µM. The proposed biosensor (GCE/MWCNT-Av/bLOx) showed a reproducibility of 6.0% and it was successfully used for determining L-lactate in food and enriched serum samples.

## 1. Introduction

The construction of electrochemical biosensors from carbon nanomaterial-based analytical platforms has received enormous attention [1,2,3]. Among these carbon nanomaterials, carbon nanotubes (CNTs) have been largely used due to their widely known properties such as high electrical conductivity, chemical stability, mechanical strength, and a high surface-to-volume ratio [4,5,6]. However, a critical aspect to take into consideration when designing these biosensors is the strategy used for the functionalization of the nanostructures that not only allows efficient exfoliation but also provides them with (bio)recognition properties [7,8,9,10,11,12,13,14]. 

It is widely known that the detection and quantification of L-lactate is relevant for clinical diagnosis, sports medicine, and the food industry [15]. The determination of L-lactate concentration in blood is essential as a diagnostics parameter due to the direct association between higher levels of L-lactate and critical health conditions (shocks, metabolic disorders, respiratory insufficiency, and heart failure). In food and beverage industries, the freshness amount of L-lactate indicates the presence of bacterial fermentation and is related to the quality of several products. Several analytical methodologies have been successfully used for lactate quantification like high-performance liquid chromatography [16], fluorometry [17], colorimetric tests [18], chemiluminescence [19], and magnetic resonance spectroscopy [20]. However, most of them present some drawbacks related to the time required for sample pretreatment, the cost of the equipment, and the requirement for trained personnel. At this scenario, electrochemical enzymatic biosensors have demonstrated to be highly important for the quantification of L-lactate due their advantageous analytical characteristics like sensitivity and selectivity, easy operation, and relative low costs [21,22]. 

L-Lactate oxidase (LOx) (EC 1.1.3.2) is a member of the family of flavoproteins and catalyzes the oxidation of L-lactate into pyruvate and generates hydrogen peroxide in the presence of oxygen [23,24]. LOx can be obtained from several bacterial sources and is used as a biocatalyst to develop enzyme-based biosensors and colorimetric *in vitro* tests for L-lactate detection [21,22,25,26]. 

The immobilization of the enzyme at the electrode surface is a crucial step to obtain electrochemical biosensors with good analytical performance. Different schemes have been proposed, among them, the avidin-biotin system allows uniform and efficient immobilization [11,27,28,29]. The pre-requisite to exploit this specific immobilization strategy is the production of biotin-tagged enzymes. Several methods have been described for the covalent attachment of biotin to proteins (a process called biotinylation). Chemical biotinylation lacks reproducibility, homogeneity, and can lead to loss of activity or alteration of the protein structure. Enzymatic biotinylation involves the use of the biotin ligase (BirA) enzyme that catalyzes the covalent attachment of biotin to the lysine within a short 15–23 amino acid peptide termed the Avi-tag. Unlike chemical biotinylation, Avi-tag biotinylation guarantees a site-specific and homogeneous modification. This type of biotinylation can be carried out *in vitro* or *in vivo*. *In vitro* biotinylation requires the production of the BirA protein and the protein of interest to be fused to the Avi-tag independently; then, *in vitro* biotinylation must be performed in the presence of biotin, Mg^2+^, and ATP. In the case of *in vivo* biotinylation, the BirA protein must be co-expressed with the protein of interest fused to the Avi-tag and biotinylation must occur while the protein is produced inside the *Escherichia coli* cell. Therefore, the protein of interest is obtained directly biotinylated after purification [29,30,31,32]. In this context, we carried out the recombinant production of a biotinylated LOx enzyme (bLOx) using an *in vivo* biotinylation approach.

In this work, a new recombinant bLOx produced in *Escherichia coli* was used for the development of an amperometric biosensor for L-lactate based on its immobilization at glassy carbon electrodes (GCEs) modified with multiwalled carbon nanotubes non-covalently functionalized with avidin (MWCNT-Av). 

Several electrochemical L-lactate biosensors have been reported for food and clinical applications in the last years. Khosravi et al. [33] reported an L-lactate biosensor using screen printed carbon electrodes modified in a more ecofriendly way, using Prussian Blue and iron oxide nanoparticles, obtained from a waste product of steel production industries, covered with polydopamine as a platform for further immobilization of LOx. Ozoglu et al. [34] proposed an enzyme-based amperometric biosensor for the determination of L-lactate produced by foodborne lactic acid bacteria isolated from fermented cheese samples. This biosensor was constructed by drop-casting a LOx solution on the Pt electrode surface using an outer layer of Nafion as an anti-interferent barrier. Madden et al. [35] reported the development of an electrochemical sensor for enzymatic L-lactate detection based on laser-scribed graphitic carbon modified with electrodeposited platinum, chitosan, and LOx. This bioplatform demonstrated potential practical applications in both saliva and human serum samples (recuperation assay ranged between 94 and 124%). Meng et al. [36] introduced a disposable flexible and skin-mountable electrode platform made of lignin-derived graphene obtained through a green route from the conversion of biomass lignin functionalized with Prussian Blue and LOx. Zhang et al. [37] proposed a disposable amperometric sensor for the quantification of L-lactate in condensed exhaled breath in healthy subject at rest and after 30 min of intense aerobic cycling exercise using a gold electrode modified with PEDOT (poly(3,4-ethylenedioxythiophene)), PSS (poly(styrenesulfonate))-Prussian Blue and LOx, making an interesting comparison between the response obtained using the commercial enzyme and another one expressed in *E. coli*. García-Morales et al. [38] constructed an L-lactate biosensor with enhanced electrochemical performance under acidic conditions by using a rationally designed mutant of lactate oxidase from *Aerococcus viridans*. This biosensor was assembled on carbon paper using chitosan as an immobilizing enzymatic agent and Prussian Blue as a mediator for its known electrochemical behavior towards H_2_O_2_. Daboss et al. [39] described a sensitive L-lactate biosensor based on screen-printed carbon electrodes modified with Prussian Blue, LOx immobilized in APTES, and with an outer perfluorosulfonated ionomer (Nafion analogue) membrane that acts as a diffusion barrier due to its intrinsic negative charge repulsing negatively charged lactate. The developed biosensor covered the range of physiological L-lactate levels in human sweat and allowed the non-invasive monitoring of hypoxia. Shitanda et al. [40] described a microfluidic system for continuously sensing L-lactate attached to a person with a design that minimizes the influence of air bubbles that may infiltrate the channel during measurements. It is based on the use of an electrode modified with thionine, followed by the incorporation of LOx and final addition of chitosan-genipin solution, which is located in a reservoir where the sweat is collected once perspiration starts. Vokhmyanina et al. [41] proposed a biosensor based on the entrapment of LOx by casting an aqueous solution of the enzyme in isopropanol containing siloxane at Prussian-Blue-modified, screen-printed carbon electrodes followed by a long period of solvent evaporation and a polycondensation process. Dagar et al. [42] proposed an amperometric biosensor obtained via covalent immobilization of LOx at a nanohybrid of chitosan, iron oxide nanoparticles, and carboxylated multiwalled carbon nanotubes electrodeposited onto a Au electrode. Deng [43] designed an impedimetric biosensor combining LOx with graphene oxide nanosheets to measure L-lactate levels in sweat with real-time response, which made it possible to monitor the physiological variations in L-lactate from resting states to peak exertion levels. Fernandes et al. [44] reported an amperometric biosensor based on carbon fiber microelectrodes modified with Pt nanoparticles and LOx to directly and continuously measure L-lactate levels during *Status epilepticus* in the rat brain (extracellular space). 

In the following sections, we present the characterization of the biomolecular recognition between the recombinant bLOx and the avidin that supports the MWCNTs, the optimization of the preparation conditions for GCE/MWCNT-Av/bLOx biosensor, and the analytical performance of the resulting GCE/MWCNT-Av/bLOx for L-lactate biosensing.

## 2. Materials and Methods

### 2.1. Reagents

Carbon nanotubes (MWCNTs; length: 2.5–20 µm; 6–13 nm of outer diameter; purity >98%), L-lactate, ascorbic acid, and uric acid were provided by Sigma-Aldrich (St. Louis, MO, USA). Ethanol HPLC grade was purchased from Cicarelli (San Lorenzo, Santa Fe, Argentina). Avidin (obtained from egg white, A-2667) was acquired from Invitrogen Life Technologies (Waltham, MA, USA). 

All the solutions were prepared using ultrapure water (resistivity = 18.2 MΩ·cm) from a Millipore-MilliQ system. The supporting electrolyte was a 0.050 M phosphate buffer solution of pH 7.40.

### 2.2. Recombinant bLOx Production

#### 2.2.1. Expression Vectors

**Figure 1:**
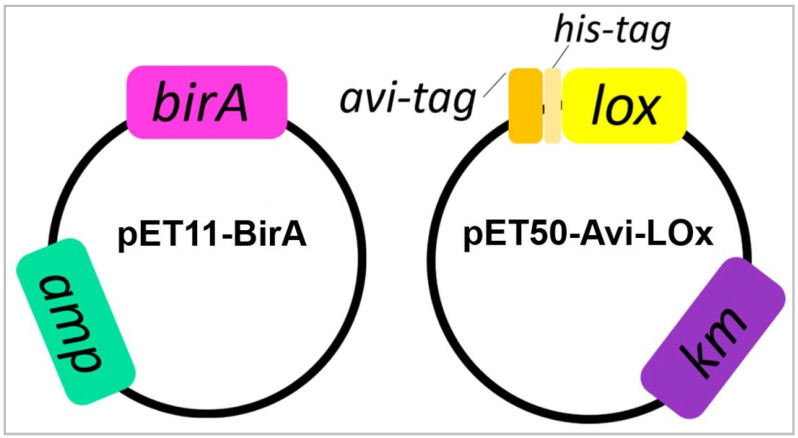
Expression vectors used to co-express the BirA enzyme and the LOx enzyme with an N-terminal Avi-tag and His-tag in *E. coli* BL21 (DE3). Pink: BirA coding sequence. Green: ampicillin resistance gene. Yellow: coding sequence of LOx fused with an N-terminal Avi-tag and His-tag. Violet: kanamycin resistance gene.

We designed two *E. coli* expression vectors (Figure 1): (1) pET11-BirA: to express the BirA enzyme that catalyzes the covalent attachment of a biotin to the Avi-tag; and (2) pET50-Avi-LOx: to overexpress the *Aerococcus viridans* LOx fused with an N-terminal Avi-tag (for biotinylation) and His-tag (for purification). For the first vector, the *E. coli* BirA coding sequence [32] was codon optimized and commercially synthesized (GenScript) and cloned into the pET11a (ampicillin resistance) plasmid. For the second one, the Avi-tag coding sequence [45] was commercially synthesized and cloned into pET-LOx (kanamycin resistance) previously obtained in our laboratory [46]. Avi-tag coding region was cloned in frame with the His-tag-LOx coding region of the pET-LOx vector to generate an N-terminal fusion (Figure 1). The pET11-BirA and pET50-Avi-LOx plasmids were sequentially inserted into the *E. coli* BL21 (DE3) strain via transformation to co-express the BirA biotin ligase and the LOx fused to the Avi-tag and His-tag. Competent *E. coli* BL21 cells were first transformed using the pET50-AviLOx vector and selected using kanamycin (25 μg mL^−1^). The strain obtained in this transformation was consecutively transformed using the pET11-BirA vector and selected using kanamycin (25 μg mL^−1^) and ampicillin (100 μg mL^−1^).

#### 2.2.2. Protein Expression and *In Vivo* Biotinylation

*E. coli* cells containing pET11-BirA and pET50-Avi-LOx vectors were grown at 37 °C with shaking in 500 mL Luria-Bertani (LB) medium supplemented with kanamycin (25 μg mL^−1^) and ampicillin (100 μg mL^−1^) to an OD_600_ of 0.6–0.8. Protein expression was induced by adding 0.4 mM isopropil β-D-1-thiogalactopyranoside (IPTG) and the medium was supplemented with 50 μM biotin, followed by incubation overnight at 20 °C with shaking [32,46]. The cells were then collected via centrifugation and a fraction of them were lysed via sonication. The lysate was centrifuged (13,000 rpm, 30 min) and the expression of biotinylated LOx protein (bLOx) in the supernatant (total soluble protein fraction) was evaluated using SDS-PAGE followed by Western blot using a streptavidin-conjugated antibody.

#### 2.2.3. Protein Purification and Activity Analysis

The cells obtained in the expression step were resuspended in 20 mL of binding buffer (50 mM Tris–HCl pH 7.5, 500 mM NaCl, 15% glycerol) and lysed via high pressure homogenization (Avestin Emulsiflex C3). The supernatants of centrifuged lysates (total soluble protein fraction) were incubated with 1 mL (bed volume) Ni-NTA agarose resin (Qiagen) at 4 °C with agitation. The unbound proteins were removed by washing them with 40 column bed volumes of binding buffer containing increased concentrations of imidazole (0, 20, 40, 60, and 80 mM). The histidine-tagged biotinylated LOx was eluted using 400 mM imidazole. The purified protein was quantified using the Bradford method using Bio-Rad Protein Assay Dye and analyzed via SDS-PAGE (15%) [46].

The non-biotinylated LOx enzyme, previously produced in our laboratory [46], was also expressed (using pET-LOx), purified and used as a control in this work. The purified bLOx was functionally evaluated. We analyzed its specific activity in comparison with the non-biotinylated LOx (without Avi-tag and biotinylation) using the activity assay described by Hiraka et al. [47].

### 2.3. Procedure and Apparatus

Electrochemical experiments were carried out using the typical system of three electrodes. Glassy carbon electrodes modified with avidin-functionalized MWCNTs (GCE/MWCNT-Av) were used for preparing the working electrodes, a wire of platinum was the auxiliary electrode, and a Ag/AgCl, 3 M NaCl was the reference electrode. The potentials indicated throughout the manuscript are referred to this latter electrode. 

Sonication was performed using a TB04TA ultrasonic cleaner (Testlab, Bernal, Argentina) of 40 kHz frequency and 160 W ultrasonic power.

Amperometric experiments were performed in a stirred 0.050 M phosphate buffer solution of pH 7.40 by applying the desired working potential (−0.100 V). All the experiments were conducted at room temperature. 

Electrochemical impedance spectroscopy (EIS) experiments were performed using a PGSTAT30 potentiostat (Methrom, Herisau, Switzerland) using 1.0 × 10^−3^ M hydroquinone/benzoquinone as a redox marker and the following working conditions—amplitude: 0.010 V, frequency range: between 1.0 × 10^−2^ and 1.0 × 10^6^ Hz, and working potential: 0.050 V. EIS spectra were analyzed using the Zview program. 

Surface plasmon resonance (SPR) experiments were accomplished using an Autolab E-SPR SPRINGLE instrument (single channel, Eco Chemie, Utrecht, The Netherlands) and a standard gold disk (BK-7 Eco Chemie) mounted on a hemicylindrical lens through index-matching oil. The platform was built on the disk previously modified via the dispersion of MWCNTs non-covalently functionalized with avidin. The solutions were injected manually and the measurements were carried out under batch conditions at 25 °C. Information about the system was obtained through the changes in the SPR minimum angle (Δθ_SPR_).

### 2.4. Preparation of GCE/MWCNT-Av/bLOx as Working Electrode

#### 2.4.1. Non-Covalent Functionalization of MWCNTs with Avidin

**Figure 2:**
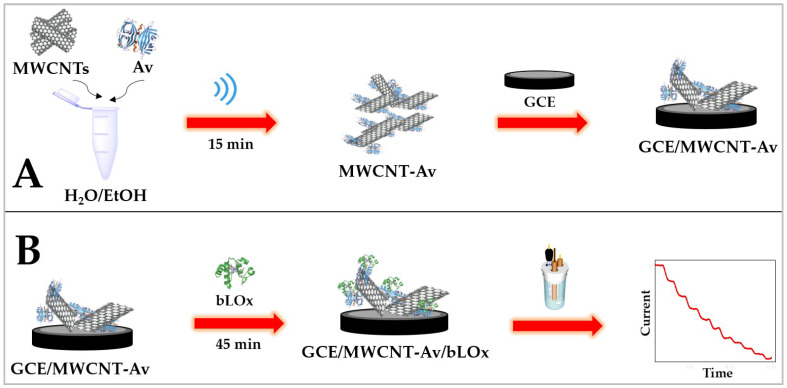
Schematic representation of the construction of the L-lactate biosensor. (**A**) Preparation of MWCNT-Av dispersion and further modification of GCE surface (GCE/MWCNT-Av). (**B**) Amperometric biosensor for L-lactate based on GCE/MWCNT-Av as a bioaffinity platform for bLOx.

MWCNTs were mixed with 1.250 mg mL^−1^ Av (prepared in 50 *v*/*v* ethanol/water) to obtain a ratio of 0.625 mg MWCNTs per mL of Av solution, and the resulting mixture was sonicated for 15 min in a sonication bath (Figure 2A).

#### 2.4.2. Construction of the Biosensor (GCE/MWCNT-Av/bLOx)

Glassy carbon electrodes were first polished using alumina slurries of 1.0, 0.3, and 0.05 µm for 2.0 min each, rinsed with MilliQ water, and dried using a nitrogen stream. The biosensor was built at the surface of the polished GCEs through deposition of 10 µL MWCNT-Av and the solvent was allowed to evaporate at room temperature (Figure 2A). 

Once washed with a 0.050 M phosphate buffer solution pH 7.40, 20 µL of 0.50 mg mL^−1^ bLOx was dropped on the top of the modified electrode for 45 min (GCE/MWCNT-Av/bLOx) (Figure 2B).

### 2.5. Determination of L-Lactate in Real Samples

Fermented milk purchased from local store. The samples were diluted with 0.050 M phosphate buffer of pH 7.40 until the desired concentration was reached. Reconstituted human serum samples (Wienner lab., Rosario, Santa Fe, Argentina) were diluted with 0.050 M phosphate buffer of pH 7.40 (1:1000). The enriched samples were obtained by adding the corresponding concentration of L-lactate to the diluted sample of serum.

## 3. Results and Discussion

### 3.1. Recombinant bLOx Production

**Figure 3:**
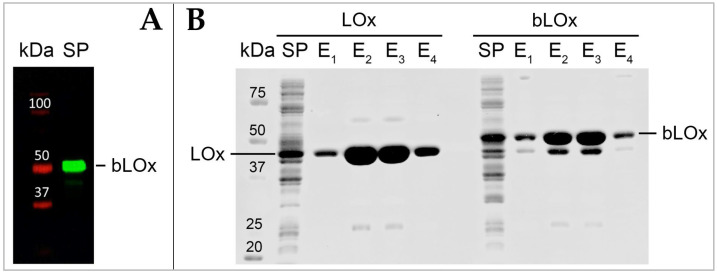
(**A**) Western blot analysis of total soluble protein fraction (SP) obtained from *E. coli* BL21 (DE3) containing pET11-BirA and pET50-Avi-LOx. Antibody: IRDye^®^ 800CW Streptavidin (LI-COR, Lincoln, NE, USA). (**B**) SDS-PAGE analysis of bLOx and LOx purification. Lane KDa: protein molecular weight marker (PageRuler Unstained Protein Ladder; Thermo Fisher Scientific Baltics UAB, Vilnius, Lithuania). Lane SP: total soluble protein fraction following cell lysis. Lanes E1–E4: elution fractions.

The bLOx expression and biotinylation was verified in total soluble protein fraction by Western blot using a streptavidin-conjugated secondary antibody. The bLOx was observed as a protein band of approximately 45–50 kDa (41 kDa LOx monomer, 4.1 kDa Avi- tag-linker-His-tag and biotin) recognized by streptavidin-conjugated that specifically binds biotin, indicating both expression and biotinylation of the bLOx enzyme (Figure 3A).

As described in the (Section 2.2.3), the recombinant histidine-tagged bLOx was purified using a Ni-NTA column. Under our experimental conditions, a high-purity bLOx was efficiently recovered after a single affinity purification step, obtaining 15–20 mg L^−1^ of bacterial culture (Figure 3B). The specific activity of purified bLOx was analyzed in comparison with a non-biotinylated LOx (without Avi-tag and biotinylation). The activity of bLOx ((102 ± 2) U mg^−1^) was approximately half that of non-biotinylated LOx activity ((216 ± 4) U mg^−1^). However, the bLOx activity obtained is within normal values compared to the activities of LOx enzymes reported in the literature and by commercial manufacturers [46,48,49,50,51,52,53,54].

### 3.2. Molecular Recognition of GCE/MWCNT-Av towards bLOx

**Figure 4:**
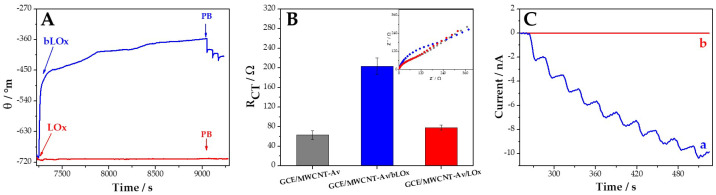
(**A**) Sensorgrams recorded after the addition of bLOx (blue line) or LOx (red line) at gold disks modified with MWCNT-Av. (**B**) Bar plots corresponding to the R_CT_ obtained at GCE/MWCNT-Av (gray bar), GCE/MWCNT-Av/bLOx (blue bar), and GCE/MWCNT-Av/LOx (red bar). Inset: Nyquist plots obtained at the corresponding electrodes. (**C**) Amperometric response for successive additions of L-lactate (100 µM) obtained at GCEs modified with MWCNT-Av/bLOx (a, blue line) or MWCNT-Av/LOx (b, red line).

As we previously mentioned, one of the goals of using avidin as a functionalization agent is to obtain nanohybrids where different biotinylated molecules could be anchored to generate diverse biosensors. In this case, we evaluated the specificity of the interaction of bLOx with the avidin that supports the MWCNTs in the MWCNT-Av nanohybrid u- sing different techniques: SPR, EIS, and amperometry. Figure 4A displays the sensorgrams obtained at gold disks modified with MWCNT-Av after the addition of 0.50 mg mL^−1^ bLOx (blue line) and LOx (red line). The amount of bLOx immobilized at MWCNT-Av-modified gold disks, calculated from the changes in the plasmon resonance angle after washing with phosphate buffer, was 2600 pg mm^−2^. On the contrary, when non-biotinylated LOx was used instead of bLOx, the amount of enzyme at Au/MWCNT-Av was negligible, confirming that bLOx is really immobilized by bioaffinity and that the interaction of bLOx at MWCNT-Av is highly specific. To reinforce this conclusion, we also performed EIS experiments. Figure 4B displays the charge transfer resistances (R_CT_) obtained for 2.0 × 10^−3^ M hydroquinone/benzoquinone at 0.050 V at GCE/MWCNT-Av (gray bar), GCE/MWCNT-Av/bLOx (blue bar), and GCE/MWCNT-Av/LOx (red bar), obtained from the Nyquist plots shown in the inset. The considerable increase in R_CT_ after the interaction of GCE/MWCNT-Av with bLOx ((2.0 ± 0.2) × 10^2^ Ω and (0.63 ± 0.09) × 10^2^ Ω for GCE/MWCNT-Av/bLOx and GCE/MWCNT-Av, respectively) and the negligible change in R_CT_ after the interaction of GCE/MWCNT-Av with LOx ((0.78 ± 0.05) × 10^2^ Ω) provide additional evidence of the highly specific interaction between MWCNT-Av and bLOx. These results allow us to confirm the successful and specific immobilization of bLOx at GCE/MWCNT-Av. To obtain information about the activity of the immobilized bLOx, we conducted amperometric experiments at −0.100 V. Figure 4C shows amperometric recordings obtained after successive additions of 100 µM L-lactate at GCE/MWCNT-Av/bLOx (blue line) and GCE/MWCNT-Av/LOx (red line). A clear and well-defined amperometric response is obtained at GCE/MWCNT-Av/bLOx, in contrast with the negligible response obtained at GCE/MWCNT-Av/LOx. In summary, SPR, EIS, and amperometric results clearly demonstrate that bLOx is successfully immobilized at MWCNT-Av and that it remains highly active.

### 3.3. Optimization of the Preparation of GCE/MWCNT-Av/bLOx

**Figure 5:**
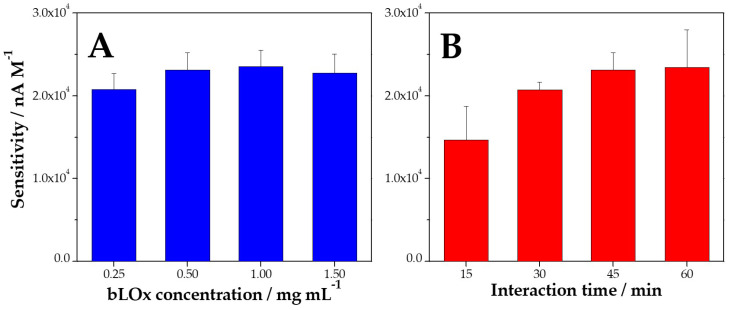
Dependence of the sensitivity to L-lactate obtained from amperometric recordings on GCE/MWCNT-Av/bLOx as a function of the bLOx concentration (**A**, blue bar) and the interaction time (**B**, red bar).

Figure 5A depicts the influence of the concentration of bLOx between 0.25 and 1.50 mg mL^−1^ on the sensitivity of the amperometric response of L-lactate at −0.100 V. This parameter did not have a significant influence on the response of the biosensor since the sensitivity slightly changed up to 0.50 mg mL^−1^ and remained almost constant thereafter. In contrast with this behavior, the interaction time between bLOx and MWCNT-Av was demonstrated to be an important parameter. In fact, the sensitivity increases between 15 and 60 min and remain almost constant for longer times (Figure 5B). Therefore, the selected conditions were 0.50 mg mL^−1^ bLOx and 45 min of interaction.

### 3.4. Analytical Performance of GCE/MWCNT-Av/bLOx for L-Lactate Biosensing

**Figure 6:**
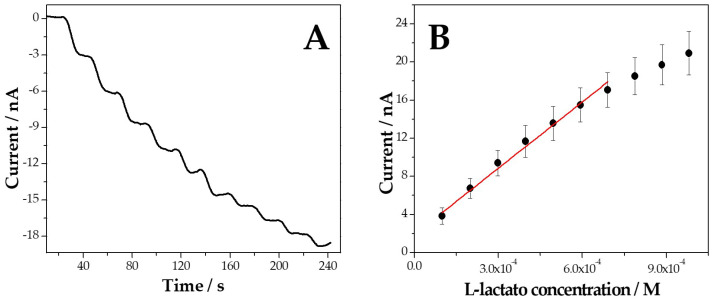
(**A**) Representative amperometric recording obtained at GCE/MWCNT-Av/bLOx after successive additions of L-lactate (1.0 × 10^−4^ M). (**B**) The corresponding calibration curve.

Figure 6A displays amperometric recordings obtained at GCE/MWCNT-Av/bLOx at −0.100 V for successive additions of 100 µM L-lactate, while Figure 6B depicts the corresponding calibration plot. A fast and well-defined response is observed after each addition of L-lactate, with a linear range from 100 and 700 µM, a sensitivity of (2.3 ± 0.2) × 10^4^ nA M^−1^ (R^2^ = 0.988), a detection limit of 33 µM (taken as 3 × standard deviation of the blank signal/sensitivity), and a quantification limit of 100 µM (taken as 10 times the ratio between the standard deviation of the blank signal and the sensitivity).

The reproducibility of GCE/MWCNT-Av/bLOx using the same dispersion and five different electrodes was 6.0%. Amperometric experiments performed in the presence of 100 µM ascorbic acid and 250 µM uric acid, demonstrated negligible interference, with 2.0% and 1.5% for ascorbic acid and uric acid, respectively, compared to the signal obtained for 125 µM L-lactate. 

The analytical application of our biosensor was evaluated using different samples. We challenged GCE/MWCNT-Av/bLOx using a commercial fermented milk and the concentration of L-lactate was 84.1 mM, showing a very good correlation with the value obtained using the enzymatic colorimetric method (r-biopharm) (93.7 mM). Recovery assays in reconstituted serum samples presented percentages of 92% and 94% for the addition of 125 µM and 250 µM L-lactate, respectively.

Finally, Table 1 compares the analytical performance of our biosensor with the most relevant electrochemical enzymatic biosensors for L-lactate reported in the last years. The detection limit obtained using GCE/MWCNT-Av/bLOx is comparable to the one reported in [38], lower than the ones reported in [33,34,35,39,40,55,56], and higher than those proposed in [36,37,41,42]. However, the biosensors with detection limits lower than our biosensor presented more complexes schemes for preparation. Zhang et al. [37] used a platform to immobilize the enzyme that involves PEDOT, PSS, and Prussian Blue. Vokhmyanina et al. [41] used a biosensor obtained after a long preparationtime of the membrane that entraps the enzyme. The biosensor proposed by Meng et al. [36] requires a long process that involves several steps to convert the lignin in a disposable L-lactate biosensor. Dagar et al. [42] proposed the use of iron oxide nanoparticles and chitosan in addition to the carbon nanostructures, with the LOx covalently attached to the nanohybrid. Therefore, our biosensor represents a competitive option for L-lactate quantification since the preparation time is short and only involves a platform of MWCNTs non-covalently functionalized with avidin and the enzyme.

## 4. Conclusions

The results described here demonstrated the successful production of an active recombinant bLOx by using an *in vivo* biotinylation strategy that allows us to obtain the biotinylated protein of interest without further processing after the purification step and, at variance with chemical biotinylation, ensures homogeneous site-specific modification. The combination of the great advantages of using this relatively cheap and ready-to-use recombinant bLOx and the versatility of the MWCNT-Av nanohybrid deposited at glassy carbon electrodes as specific anchoring site made the development of a sensitive and selective amperometric L-lactate biosensor with successful application for the quantification of L-lactate in real samples possible. The high efficiency of this bioanalytical platform for L-lactate sensing opens the doors to new designs that involve wearable biosensors with mass production capability and portability, which could be used in the food industry, biotechnology, and clinical care.

## Figures and Tables

**Table 1:** Comparison of electrochemical techniques, platforms, and sensing performance of LOx-based biosensor for L-lactate reported in the last years.

Technique	Electrode	Platform	Linear Range (mM)	Detection Limit (mM)	Ref.
Amperometry	SPCE	PB/Fe_3_O_4_@PDA-LOx	0.1–4.62	0.32	[33]
Amperometry	Pt	LOx/Nafion	50–350	31	[34]
Amperometry	LSG	Pt/CHIT/LOx	0.2–3.0	0.11	[35]
Amperometry	SPCE	rLIG/LOx	2–16	0.007	[36]
Amperometry	SPAuE	PEDOT:PSS-PB-LOx_(com)_	ND	0.0000783	[37]
		PEDOT:PSS-PB-LOx_(exp)_		0.000465	
Amperometry	CP	PB-Av-bLOx	0.2–2	0.038	[38]
Amperometry	SPCE	PB/LOx/PFSI	1–100	1	[39]
Chronoamperometry	SPCE	Thionine-methanol/LOx/CHIT	1–10	1	[40]
Amperometry	SPCE	APTMS-MAPS/LOx-PB	0.001–1	0.0005	[41]
Amperometry	AuE	LOx/CHIT/Fe_3_O_4_NPs/cMWCNT	0.112–0.183	0.0006	[42]
EIS	GO nanosheets	PANHS/LOx	1–80	1	[43]
Amperometry	CFM	Pt/Nafion-LOx	0.05–0.5	-	[44]
DPV	SPCE	LOx/G-PU-rGO-PB	5–25	0.4	[55]
Amperometry	SPCE	ISF/PB-LOx	1.0–5.0	0.15	[56]
Amperometry	GCE	MWCNT-Av/bLOx	0.100–0.700	0.033	This work

Abbreviations: SPCE: screen-printed carbon electrodes; PB: Prussian Blue; Fe_3_O_4_@PDA: iron oxide nanoparticles coated with polydopamine; LOx: lactate oxidase; LSG: laser-scribed graphitic carbon electrode; CHIT: chitosan; rLIG: reduced laser-induced graphene; SPAuE: screen-printed gold electrode; PEDOT: poly(3,4-ethylenedioxythiophene); PSS: poly(styrenesulfonate); LOx_(com)_: commercial lactate oxidase; LOx_(exp)_: expressed lactate oxidase; ND: no data; CP: carbon paper; Av: avidin; bLOx: biotinylated lactate oxidase; PFSI: perfluorosulfonated ionomer; APTMS: (3-aminopropyl)trimethoxysilane; MAPS: trimethoxy [3-(methylamino)propyl]silane; AuE: gold electrode; Fe_3_O_4_NPs: iron oxide nanoparticles; cMWCNT: carboxylated multiwalled carbon nanotubes; EIS: electrochemical impedance spectroscopy; GO: graphene oxide; PANHS: 1-pyrenebutyric acid N-hydroxysuccinimide ester; CFM: carbon fiber microelectrode; DPV: differential pulse voltammetry; G-PU: graphite-polyurethane; rGO: reduced graphene oxide; ISF: collection of interstitial fluid; GCE: glassy carbon electrode; MWCNT: multiwalled carbon nanotubes.

## Data Availability

Data are contained within the article.
